# Altered expression of membrane-bound and soluble CD95/Fas contributes to the resistance of fibrotic lung fibroblasts to FasL induced apoptosis

**DOI:** 10.1186/1465-9921-6-37

**Published:** 2005-04-17

**Authors:** Frank Bühling, Aline Wille, Christoph Röcken, Olaf Wiesner, Anja Baier, Ingmar Meinecke, Tobias Welte, Thomas Pap

**Affiliations:** 1 Institute of Immunology, Otto-von-Guericke-University, Magdeburg, Germany; 2 Division of Experimental Rheumatology, Otto-von-Guericke-University, Magdeburg, Germany; 3 Institute of Pathology, Otto-von-Guericke-University, Magdeburg, Germany; 4 Department of Pneumology, Hannover Medical School, Hannover, Germany; 5 Division of Molecular Medicine of Musculoskeletal Tissue, University Hospital, Munster, Germany; 6 Institute of Clinical Chemistry and Laboratoy Diagnostics, Carl-Thiem-Klinikum Cottbus gGmbH, Thiemstr. 111, 03048 Cottbus, Germany

## Abstract

**Background:**

An altered susceptibility of lung fibroblasts to Fas-induced apoptosis has been implicated in the pathogenesis of pulmonary fibrosis; however, the underlying mechanism is not completely understood. Here, we studied the susceptibility of lung fibroblasts, obtained from patients with (f-fibs) and without pulmonary fibrosis (n-fibs), to FasL- (CD95L/APO-1) induced apoptosis in relation to the expression and the amounts of membrane-bound and soluble Fas. We also analysed the effects of tumor necrosis factor-β on FasL-induced cell death.

**Methods:**

Apoptosis was induced with recombinant human FasL, with and without prior stimulation of the fibroblasts with tumor necrosis factor-α and measured by a histone fragmentation assay and flow cytometry. The expression of Fas mRNA was determined by quantitative PCR. The expression of cell surface Fas was determined by flow cytometry, and that of soluble Fas (sFas) was determined by enzyme-linked immunosorbent assay.

**Results:**

When compared to n-fibs, f-fibs were resistant to FasL-induced apoptosis, despite significantly higher levels of Fas mRNA. F-fibs showed lower expression of surface-bound Fas but higher levels of sFas. While TNF-α increased the susceptibility to FasL-induced apoptosis in n-fibs, it had no pro-apoptotic effect in f-fibs.

**Conclusions:**

The data suggest that lower expression of surface Fas, but higher levels of apoptosis-inhibiting sFas, contribute to the resistance of fibroblasts in lung fibrosis against apoptosis, to increased cellularity and also to increased formation and deposition of extracellular matrix.

## Background

Lung fibrosis is the final common and often irreversible pathway of different lung diseases, such as idiopathic interstitial pneumonitis (idiopathic pulmonary fibrosis) and granulomatous diseases (sarcoidosis) [1-3]. Though these diseases are different in their etiology, all are characterized by zones of lung injury where varying numbers of fibroblasts proliferate and contribute to the accumulation of extracellular matrix (ECM). Interstitial and intralumal deposition of connective tissue then disrupts the lung architecture and impairs respiratory function.

Recent studies have shown that the development of lung fibrosis is accompanied by the differentiation of normal lung fibroblasts into myofibroblasts. These myofibroblasts express α-smooth muscle actin, and they are thought to be the major source of collagen and profibrogenic growth factors in the fibrosing lung [4]. Additionally, decreased apoptosis of these cells may contribute to the remodeling of lung tissue during chronic inflammation. Apoptosis is a physiological process that is highly selective in eliminating aged and injured cells. In addition to internal pathways that mainly trigger apoptosis in response to cytotoxic stress, apoptosis can also be induced by cell-membrane-anchored signaling pathways of the TNF-superfamily: the CD95-receptor/CD95-ligand-system (Fas/FasL or APO-1) and the tumor necrosis factor (TNF)-related apoptosis inducing ligand (TRAIL or APO-2L) with the TRAIL receptors 1 and 2 (TRAIL-R1 and R2) and the decoy receptors DcR1 (TRAIL-R3) and DcR2 (TRAIL-R4). TRAIL induces programmed cell death in many tumor cells, but not in normal, non-neoplastic cells [5].

The mechanisms through which stimulation of Fas by FasL initiate apoptosis have been extensively investigated. It is also known that mesenchymal, fibroblast-like cells express Fas. Alterations in the susceptibility of these cells to Fas-induced cell death contribute to the pathogenesis of lung fibrosis, [6,7] and myofibroblasts are susceptible to the suppression of apoptosis by transforming growth factor-β1 (TGF-β1) [6] and resistant to interleukin (IL)-6-induced apoptosis [8]. However, the molecular mechanisms which regulate these alterations in resistance to proapoptotic signals, and thus contribute to decreased apoptosis of fibroblasts during chronic inflammation, are not known in detail.

Apoptosis is regulated by a complex system consisting of numerous proteins and cascading proteolytic and phosphorylation steps. The contribution of isolated elements of the system to the regulation of apoptosis resistance is less well characterized. The binding of soluble or cell surface bound FasL with surface Fas may initiate apoptosis. Consequently, the intensity and the stochiometry of the Fas-FasL interaction could play a crucial role in the regulation of apoptosis.

In this study we systematically investigated the expression and interplay of the Fas/FasL system in fibroblasts obtained from patients with and without lung fibrosis. We aimed to clarify the possible involvement of the Fas/FasL system in the survival of lung myofibroblasts and the development of lung fibrosis.

## Methods

### Human tissues

Tissue samples from patients with (*n *= 5) and without lung fibrosis (*n *= 6) were obtained from diagnostic open lung biopsies (fibrotic samples) and from healthy tissue areas during pneumonectomy for tumor resection (non-fibrotic samples). The fibrotic samples comprised the following diseases: usual interstitial pneumonia (UIP, two patients), non-specific interstitial pneumonia (NSIP, one patient), bronchiolitis obliterans-organizing pneumonia (BOOP, two patients) [3]. Although these diseases are different in clinical behavior and outcome, they are characterized by increased amounts of activated fibroblasts and increased matrix deposition. The histology of these tissue samples was recently partially described [9]. The lung fibroblasts were compared to synovial fibroblasts (n = 5), which were obtained from patients with osteoarthritis during joint replacement surgery. An experienced surgical pathologist (C.R.) examined the tissue specimens. All tissue samples were obtained immediately after surgery and used for the isolation of fibroblasts. The tissue sampling was approved by the local ethical committee.

### Characterization of fibroblasts by flow cytometry

Fibroblasts were trypsinized. For extracellular staining, fibroblast-specific antibodies [clones AS02 (anti-Thy-1) and D7-Fib; Dianova, Hamburg, Germany], a macrophage-specific anti-CD68 (clone KP1; Signet Laboratories, Inc., Dedham, MA) and a pan-leukocyte anti-CD45 antibody were used.

Cells were incubated with the primary antibodies for 30 min and with a fluorescein isothiocyanate (FITC)-labeled goat-anti-mouse IgG for 20 min. In addition, intracellular fluorescence staining was performed with anti-prolyl-4-hydroxylase antibodies (clone 5B5; DPC Biermann, Bad Nauheim, Germany) using the Fix and Perm reagent (Dianova) according to the instructions of the manufacturer. For the analysis, a FACSCalibur (Becton Dickinson, Heidelberg, Germany) flow cytometer was used.

### Cell culture

Fibroblasts were obtained by mincing freshly excised lung parenchyma into ~1 mm^3 ^pieces, followed by digestion with collagenase IV (1 mg/ml, Sigma, Deissenhofen, Germany) for 30 min at 37 °C. Fibroblasts were cultured in a 75-ml tissue culture flask containing Iscove's modified Dulbecco's medium with 10% (w/v) fetal calf serum (FCS), 10^-3 ^M glutamine and antibiotics, at 37°C and 5% (v/v) CO_2 _until they reached confluence. Only fibroblasts between passages 3 and 8 were used for the experiments.

### Determination of collagen and ECM deposition

Collagen secretion and deposition into the ECM was assessed by proline incorporation assays originally developed by Peterovsky and Diegelmann [10] and described in detail earlier [11,12]. All assays were performed in triplicate. Briefly, 5 × 10^4 ^fibroblasts were seeded into 24-well plates (Falcon, Heidelberg, Germany) in culture medium containing 10 % FCS. After 16 h, the medium was changed to low serum medium (Dulbecco's modified Eagle's medium supplemented with 0.1% FCS, 100 μg/ml L-ascorbic acid) containing [2,3,4,5-^3^H]-lL-proline (2 μCi/ml, NEN, Boston, MA). When indicated, E64d was added (10 μM). After 72 h, the culture medium was removed and the remaining fibroblasts were lysed with distilled water (10 min, room temperature). The ECM was ethanol fixed (70% ethanol, 15 min, RT). Half of the wells were incubated with 30 U/ml collagenase (*Clostridium histolyticum*, Sigma, Deissenhofen, Germany) in collagenase assay buffer (50 mM Tris-HCL, pH 7.5, 5 mM CaCl_2_, 2.5 mM *N*-ethylmaleimide) for 4 h at 37°C. The remaining wells were incubated with assay buffer. The supernatants were removed and residual ECM was solubilized by overnight incubation in 0.3 M NaOH-1% SDS. Equal numbers of aliquots of supernatants obtained after collagenase digestion and supernatants containing the residual ECM were subjected to liquid scintillation counting. The counts measured in supernatants after collagenase treatment represent the collagen content. The amount of [^3^H]proline measured after solubilization of the remaining ECM represents non-collagenous ECM. The total of both counts was equal to the counts from solubilized ECM without collagenase treatment and represents the total proline incorporation. Relative ECM synthesis can be calculated by the established formula [12]: ECM = CPM in collagen + (5.4 × CPM in non-collagen ECM). The formula contains the factor 5.4 to correct for the 5.4-fold higher proline or hydroxyproline content of collagens compared with that of other proteins.

### Induction and detection of apoptosis

Fibroblasts were stimulated with 100 ng/ml recombinant human FasL for 16 h as described [13]. When indicated, cells were preincubated with TNF-α or cycloheximide (100 μg/ml) for 24 h. Subsequently, apoptosis was determined using a histone fragmentation assay (Cell Death Detection ELISAPlus, Roche Diagnostics, Mannheim, Germany) according to the manufacturer's instructions. This assay is based on a quantitative sandwich-enzyme-immunoassay using mouse monoclonal antibodies against DNA and histones that allow for the specific, quantitative determination of cytoplasmatic histone-associated-DNA-fragments (mono- and oligonucleosomes) in the cell lysates. The ELISA plates were read at 405 nm (490 nm reference). We have shown before that the results obtained using this assay correlate to the amount of apoptotic cells found after TUNEL staining [14].

Additionally, apoptosis was measured in lung fibroblasts using TUNEL staining (ApoBrdU kit, Pharmingen, Heidelberg, Germany). Briefly, following induction of apoptosis, cells were fixed in 1% paraformaldehyde and incubated with Br-dUTP in the presence of TdT enzyme, which results in the incorporation of Br-dUTP into exposed 3-OH DNA ends. Br-dUTP sites were then labeled with FITC-conjugated anti-Br-dUTP antibodies. The number of apoptotic cells was measured using flow cytometry (FACS Calibur, Becton Dickinson), and labeling with Br-dUTP was compared with that of unstimulated controls.

### Measurement of Fas/CD95 mRNA

Expression levels of Fas/CD95 mRNA were analyzed by quantitative real time PCR using a fluorogenic 5'-nuclease assay (TaqMan©, Applied Biosystems, Weiterstadt, Germany) on a ABI Prism 7900 HT Sequence Detection system. For each experiment, total RNA was extracted from 10^5 ^cells using the RNeasy system (Qiagen, Hilden, Germany). Total RNA was reverse transcribed using random hexamer primers. For quantitative PCR, the appropriate primers and FAM-TAMRA labeled probes were purchased as 20-fold concentrated predeveloped assays from Applied Biosystems and used according to the instructions of the manufacturer. 18S rRNA gene was co-amplified as an internal standard. Data were calculated with the ΔΔCt method as described [15].

### Measurement of soluble Fas (sFas) in cell culture supernatant and cell surface bound Fas

For the detection of sFas in the cell culture supernatants of the fibroblasts, a commercially available ELISA (Quantikine Assays, R&D Systems, Wiesbaden, Germany) was used according to the manufacturer's instructions.

The amount of cell-surface-bound Fas was measured by flow cytometry after staining the fibroblasts with FITC-labelled anti-Fas/CD95 antibodies (Becton Dickinson).

### Statistical analysis

All statistical analyses were performed with SPSS 10.0 for Windows (SPSS, Chicago, IL). Results were presented as mean values ± SE. Mean values were compared by Student's t-Test. In addition the data were analysed using the non-parametric Mann-Whitney-U-Test. Differences were considered to be significant if the *p*-values were below 0.05 in both tests.

## Results

### Characterization of lung fibroblasts

**Figure 1:**
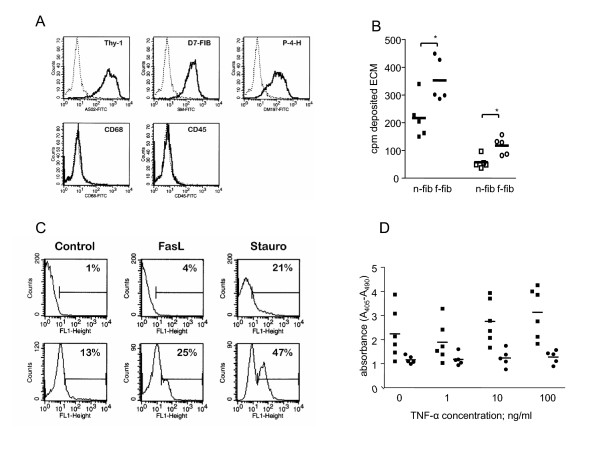
**Lung fibroblasts from patients with fibrotic lung diseases are resistant to apoptosis. *A: ***Characterization of isolated fibroblasts using flow cytometry with lineage-specific monoclonal antibodies. Immunoreactivity was detected after staining with the fibroblasts specific anti-Thy-1, D7-fib and anti-P-4-H antibodies, but not with anti-CD45 and anti-CD68 antibodies. The specificity of the immunostaining (solid line) was tested using irrelevant isotype controls (dotted line). All fibroblasts samples were analyzed and representative figures were presented. ***B: ***Matrix deposition by isolated fibroblasts as determined by a [^3^H]proline incorporation. Fibroblasts derived from patients with lung fibrosis deposited more extracellular matrix (solid circles) and collagen (open circles) than non-fibrotic fibroblasts (solid and open squares). ***C: ***Increased apoptosis in lung fibroblasts (upper panel) compared to synovial fibroblasts (lower panel). Apoptosis was induced by incubation with Fas ligand (rhFasL) and measured after TUNEL staining. Lung fibroblasts showed significantly more apoptosis resistance. ***D: ***Increased resistance to pro-apoptotic signals in fibrotic fibroblasts (square) compared to control fibroblasts (circle). Apoptosis was induced by rhFasL after pre-incubation with TNF-alpha. Apoptosis was measured by quantification of histone-bound DNA fragments.

The fibroblasts isolated from lung tissue specimens were characterized with respect to the expression of lineage-specific marker proteins (Fig. 1A). The majority of the cells stained with antibodies directed against Thy-1, an antigen that is specific for fibroblasts. These cells also expressed the fibroblast specific antigen D7-Fib and the enzyme prolyl-4-hydroxylase, which is involved in collagen synthesis. Neither CD68, a marker of monocytes/macrophages, nor CD45, a leukocyte membrane protein, were detected. We found no significant differences in the phenotypic characteristics of fibroblasts which were derived from different patient groups.

The matrix production of fibroblasts isolated from fibrotic (fibrotic fibroblasts, f-fibs) and non-fibrotic lung tissues (normal fibroblasts, n-fibs) was also analyzed: F-fibs produced significantly more ECM proteins, including collagen, than n-fibs (Fig. 1B) independent from the underlying disease.

### Resistance to Fas induced apoptosis in lung fibroblasts

Recently we showed that fibroblasts derived from patients with different inflammatory joint diseases display different susceptibilities to FasL-induced apoptosis [14]. Comparing fibroblasts derived from human lung tissues, we applied the same conditions for induction of apoptosis and found that lung fibroblasts are generally more resistant to FasL-induced apoptosis than synovial fibroblasts (Fig. 1C). Similar results were found after staurosporin treatment of fibroblasts (Fig. 1C) and after induction of apoptosis by anti-Fas antibodies (not shown). The percentage of apoptotic cells, as determined by TUNEL staining, was at the detection limit. Therefore this method could not be used for the comparison of apoptosis in lung fibroblasts. On the other hand, we found low but measurable apoptosis after quantification of the amount of histone-associated DNA fragments in the cellular supernatants. Comparison of f-fibs and n-fibs confirmed that f-fibs were more resistant to FasL-induced apoptosis. Incubation of the cells with TNF-α slightly increased the susceptibility of n-fibs to apoptosis, but it had no affect on apoptosis in f-fibs (Fig. 1D).

Recently Tanaka et al. have shown that the resistance to anti-Fas-induced apoptosis in lung fibroblasts is mediated by the overexpression of the specific inhibitors of apoptosis X-chromosome-linked inhibitor of apoptosis (ILP) and FLICE-like inhibitor protein (FLIP) [16].

**Figure 2:**
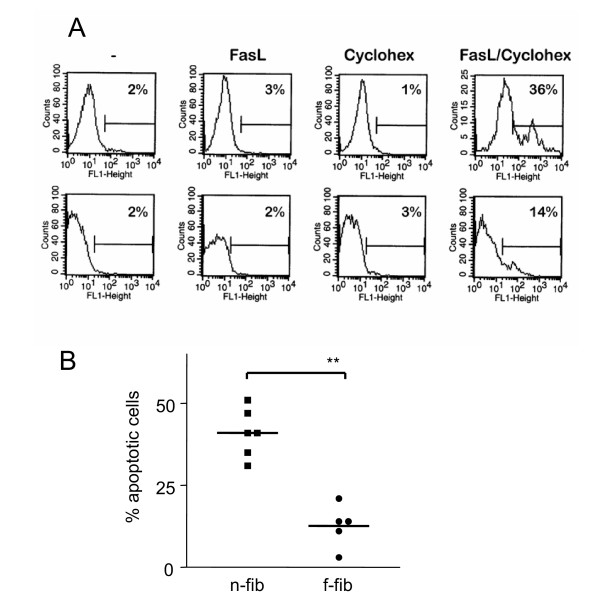
**Resistance to pro-apoptotic signals in fibrotic fibroblasts after incubation with cycloheximide and Fas ligand. *A: ***Representative histograms of non-fibrotic (upper panel) and fibrotic fibroblasts (lower panel). The cells were incubated with medium, FasL, cycloheximide or FasL+cycloheximide. Only the incubation with FasL and cycloheximide resulted in significant amounts of apoptotic cells. ***B: ***Fibrotic fibroblasts (circles) showed increased resistance to the induction of apoptosis by FasL and cycloheximide in comparison to non-fibrotic fibroblasts (squares). Apoptotic cells were detected by flow cytometry after TUNEL staining. The cumulative data of all samples are represented as mean ± SEM, **p < 0.01.

Those authors found that suppression of protein synthesis using cycloheximide decreased the concentration of these short-lived inhibitory proteins and led to increased susceptibility to Fas-mediated apoptosis. We used cycloheximide to analyse whether the differences in sensitivity to FasL-induced apoptosis depended on different expression levels of short-lived inhibitory proteins. As expected, preincubation of fibroblasts with cycloheximide increased apoptosis as determined by TUNEL-staining. However, the percentage of apoptotic cells among f-fibs was still lower than among n-fibs (Fig. 2), which suggests that short-lived inhibitory proteins do not contribute to the difference in apoptosis resistance between n-fibs and f-fibs. Therefore, other mechanisms are involved in the regulation of the resistance to FasL-induced apoptosis in f-fibs.

### Expression of Fas

**Figure 3:**
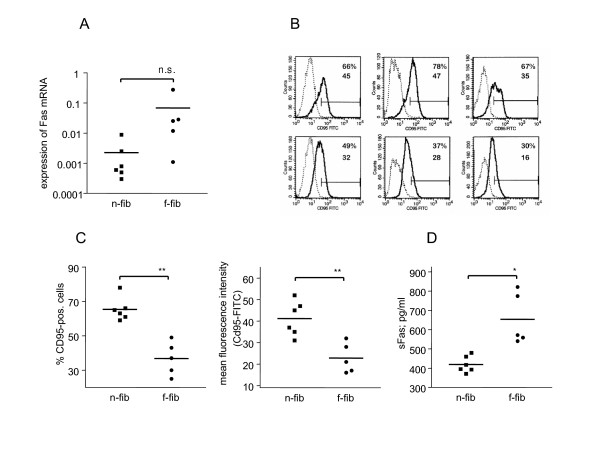
**Expression of soluble and surface-bound Fas in normal and fibrotic lung fibroblasts. *A: ***Increased levels of Fas-mRNA were found in fibrotic fibroblasts (circles) in comparison to non-fibrotic fibroblasts (squares). Fas-mRNA levels were measured using quantitative RT-PCR. ***B: ***Fas surface expression on isolated fibroblasts. Representative histograms of Fas-immunostaining on isolated fibroblasts (solid line). The specificity of the immunostaining was shown using irrelevant isotype-matched control antibodies (dotted line). Non-fibrotic fibroblasts (upper panel) expressed more surface bound Fas than fibrotic fibroblasts (lower panel). ***C: ***Cumulative data of all analysed samples showed that the percentages of Fas-positive cells (left panel) as well as the mean fluorescence intensities (right panel) were lower in fibrotic fibroblasts (circles) compared to non-fibrotic fibroblasts (squares). The results are represented as mean ± SEM. ***D: ***Increased concentration of soluble Fas in the supernatant of fibrotic fibroblasts (circles) in comparison to non-fibrotic fibroblasts (squares). The concentration of soluble Fas was measured by ELISA. The results are represented as mean ± SEM, *p < 0.05, **p < 0.01.

Expression of Fas plays a crucial role in FasL-induced apoptosis. Therefore, we investigated Fas-mRNA expression using quantitative RT-PCR analysis. Fas-mRNA levels were normalized to 18S-ribosomal RNA. Surprisingly, we found increased expression of Fas-mRNA in f-fibs (Fig. 3A). To determine whether increased Fas mRNA in f-fibs translates into increased levels of cell surface Fas, we used flow cytometry to analyze the expression of Fas on the cell surface of lung fibroblasts. We found that 65 ± 3% (mean fluorescence intensity 41 ± 5%) of n-fibs and 41 ± 5% of f-fibs (mean fluorescence intensity 24 ± 2%) expressed Fas at the cell surface (Fig. 3B, C). Based on these data, we then determined the concentration of soluble Fas in the culture supernatant and found an increased concentration of soluble Fas in the supernatant of f-fibs (Fig. 3D).

## Discussion

Lung fibrosis remains a devastating clinical condition with very limited therapeutic options. A number of experimental approaches have been investigated in clinical trials, including the modulation of key cytokines and growth factors, and treatment with corticosteroids or immunosuppressants. In a number of patients, especially those with UIP, these treatments have little effect on patient outcome [17-19]. The persistence of fibrotic lesions, which characterize lung fibrosis and lead to organ dysfunction, suggests that decreased apoptosis of myofibroblasts may play a major role in the pathology of lung fibrosis.

The present study provides evidence that fibroblasts derived from lung tissues of patients with lung fibrosis are characterized by a relative resistance to Fas-mediated apoptosis. In this context we have shown that the resistance to apoptosis depends not only on the expression of short-lived intracellular anti-apoptotic proteins, but that increased production of soluble Fas adds to this process. The resulting long-lived cells may contribute to increased matrix-deposition, and thus to altered tissue remodeling in the diseased lung.

In our study we used fibroblasts from patients with different fibrotic lung diseases, which were characterized by a 1.6-fold increase in production of extracellular matrix proteins, particularly collagen. The findings suggest that these cells retained their fibrotic differentiation state in short-term culture. Previously, it was shown that lung fibrosis is characterized by predominant differentiation of fibroblasts into myofibroblasts [20]. These cells are characterized by increased ECM production. The data concerning apoptosis in these cells are conflicting. Whereas Ramos and coworkers reported increased spontaneous apoptosis in fibroblasts obtained from patients with UIP, [20] TANAKA et al. found a high resistance to Fas-mediated apoptosis in lung fibroblasts [16]. In addition, it was reported that the apoptosis of myofibroblasts is suppressed by TGF-β1 [6] and that increasing amounts of TGF-β1 are produced by f-fibs [20]. In our experiments we found very low levels of apoptosis in all the lung fibroblasts investigated. After TUNEL-staining, the spontaneous amount of apoptotic cells was generally below 2%. This was similar to the data derived from synovial fibroblasts [14]. However, in contrast to synovial fibroblasts, apoptosis remained low after incubation of lung fibroblasts with FasL, anti-Fas antibodies or staurosporin. The data are consistent with the findings of Tanaka et al. who investigated normal lung fibroblasts and the lung fibroblast cell line WI-38 [16]. Only the application of very sensitive detection systems allowed us to quantify apoptosis in these cells. Using these assays we were able to demonstrate that f-fibs are more resistant to Fas-mediated apoptosis than n-fibs. However, we found no direct correlation between the matrix production of isolated fibroblasts and the amount of apoptosis.

Apart from interfering with receptor activation at the cell-surface, apoptosis can also be blocked by intracellular anti-apoptotic proteins. For example FLICE-like inhibitory proteins (FLIPs) can prevent the recruitment and activation of caspase 8 (FLICE) to the Fas-associated protein with death domain (FADD), and thus inhibit the formation of the death inducing signaling complex (DISC). In addition, anti-apoptotic members of the bcl-family, e.g., Bcl-2, MCL-1 and A1, prevent the mitochondrial cytochrome c release. Recently, the inhibitor of apoptosis (IAP) family of genes was identified [21]. The X-linked IAP (ILP) suppresses apoptosis by direct inhibition of caspase 3. In a variety of experimental systems it has been shown that the overexpression of these anti-apoptotic proteins results in resistance to pro-apoptotic signals [21-23]. Therefore, it was tempting to speculate that a differential expression of these anti-apoptotic proteins in n-fibs and f-fibs may cause the resistance to Fas-mediated apoptosis in lung fibroblasts.

Anti-apoptotic bcl-2 proteins and IAPs are characterized by a very short half-life. [22,24]. Cycloheximide, which blocks protein synthesis, was shown to decrease the concentration of ILP and FLICE in human lung fibroblasts on the one hand, and to increase the sensitivity of these cells to Fas-mediated apoptosis on the other hand [16]. Our experiments showed that short-lived anti-apoptotic proteins are generally involved in the apoptosis resistance of lung fibroblasts. However, they did not contribute to the different susceptibilities of n-fibs and f-fibs. Finally, we found a difference in the Fas-mRNA levels. F-fibs had higher Fas-mRNA levels with lower levels of surface-bound Fas-receptor than n-fibs. At the same time, f-fibs exhibited higher expression of soluble Fas, which exerts an anti-apoptotic function [25]. It has been shown that soluble Fas is produced as an alternatively spliced variant of Fas. On the other hand increased soluble Fas concentrations were found in patients with rheumatoid arthritis and the release was correlated to increased activities of matrix metallo proteases [26]. We have recently shown that fibrotic fibroblasts expressed increased amounts of the potent protease cathepsin K [9]. An important role of matrix metalloproteases was shown by other groups [27,28]. In summary the release of soluble Fas can be regulated by different mechanisms. Part of them is activated in fibrotic fibroblasts.

From these data, we conclude that the increased resistance to pro-apoptotic signals in lung fibroblasts obtained from patients with fibrosis is mediated at least in part by increased amounts of soluble Fas.

## Authors' contributions

FB cultured the fibroblasts, drafted the manuscript and participated in the design of the study. AW measured apoptosis by flow cytometry. AB performed quantitative RT-PCR analyses. IM carried out sFas analyses and cells death assays. OW did the bronchoscopy and tissue biopsies. CR carried out the histo-morphological classification of tissue samples. TW conceived the study, participated in the design of the study and coordinated the tissue sampling. TP conceived the study, established quantitative RT-PCR and helped to draft the manuscript. All authors read and approved the manuscript.
